# Genome-Wide Identification and Drought-Responsive Functional Analysis of the *GST* Gene Family in Potato (*Solanum tuberosum* L.)

**DOI:** 10.3390/antiox14020239

**Published:** 2025-02-19

**Authors:** Ningfan Shi, Youfang Fan, Wei Zhang, Zhijia Zhang, Zhuanfang Pu, Zhongrun Li, Lijun Hu, Zhenzhen Bi, Panfeng Yao, Yuhui Liu, Zhen Liu, Jiangping Bai, Chao Sun

**Affiliations:** College of Agronomy/State Key Laboratory of Aridland Crop Science, Gansu Agricultural University, Lanzhou 730070, China; shinf@st.gsau.edu.cn (N.S.); fanyf@st.gsau.edu.cn (Y.F.); 1073323120531@st.gsau.edu.cn (W.Z.); 1073323020278@st.gsau.edu.cn (Z.Z.); puzf@st.gsau.edu.cn (Z.P.); 1073324020399@st.gsau.edu.cn (Z.L.); 1073324020290@st.gsau.edu.cn (L.H.); bizz@gsau.edu.cn (Z.B.); yaopf@gsau.edu.cn (P.Y.); lyhui@gsau.edu.cn (Y.L.); liuzhen@gsau.edu.cn (Z.L.); baijp@gsau.edu.cn (J.B.)

**Keywords:** potato, glutathione S-transferase, drought stress, DNA demethylation

## Abstract

Glutathione S-transferases (*GSTs*) play crucial roles in crop stress tolerance through protection against oxidative damage. In this study, we conducted genome-wide identification and expression analysis of the *GST* gene family in the autotetraploid potato cultivar Cooperative-88 (C88) using bioinformatic approaches. We identified 366 *GST* genes in the potato genome, which were classified into 10 subfamilies. Chromosomal mapping revealed that *StGSTs* were distributed across all 12 chromosomes, with 13 tandem duplication events observed in three subfamilies. Analysis of protein sequences identified 10 conserved motifs, with motif 1 potentially representing the *GST* domain. Analysis of cis-acting elements in the *StGSTs* promoter regions suggested their involvement in stress response pathways. RNA-seq analysis revealed that most *StGSTs* responded to both drought stress and DNA demethylation treatments. Quantitative PCR validation of 16 selected *StGSTs* identified four members that showed strong responses to both treatments, with distinct expression patterns between drought-tolerant (QS9) and drought-sensitive (ATL) varieties. Transient expression assays in tobacco demonstrated that these four *StGSTs* enhanced drought tolerance and may be regulated through DNA methylation pathways, though the precise mechanisms require further investigation. These findings provide a theoretical foundation for understanding the response and epigenetic regulation of potato *GST* genes under drought stress.

## 1. Introduction

Plants exposed to abiotic stresses, including drought, salinity, and temperature fluctuations, accumulate reactive oxygen species (ROS), leading to oxidative damage of cellular membranes, biological macromolecules, and ultimately, cell death [1]. To counter these stresses, plants employ various enzymatic and non-enzymatic defense systems [2,3]. Glutathione S-transferase (GST) represents a crucial superfamily of enzymes that mitigate oxidative damage by eliminating excess ROS, thereby protecting cellular membrane integrity and protein function [4].

Glutathione S-transferase (GST, EC 2.5.1.18), an ancient enzyme superfamily present across prokaryotes and eukaryotes, primarily catalyzes the conjugation of reduced glutathione (γ-Glu-Cys-Gly, GSH) to electrophilic substrates, forming more soluble products [5,6,7]. Crystallographic studies reveal that *GSTs* typically exist as dimers composed of homologous or heterologous subunits, with each subunit comprising two domains: an N-terminal domain containing GSH-binding sites and a C-terminal domain housing electrophilic substrate binding sites [1,8,9]. *GSTs* exhibit remarkable functional diversity, participating in detoxification, metabolism, transport, and chelation of both endogenous and xenobiotic compounds. Additionally, *GSTs* function in ROS detoxification, neutralizing superoxide radicals, hydroxyl radicals, alkoxy radicals, hydrogen peroxide, and singlet oxygen generated in chloroplasts and mitochondria [10].

Plant *GSTs* comprise 14 distinct classes, as revealed by phylogenetic analyses of sequences from *Arabidopsis thaliana*, *Hordeum vulgare* L., *Oryza sativa* L., *Pinus tabuliformis Carr*., *Populus trichocarpa*, and *Solanum lycopersicum*. These classes include phi (F-type), tau (U-type), theta (T-type), zeta (Z-type), lambda (L-type), hemerythrin, iota (I), ure2p, GHR (glutathione hydroquinone reductase), EF1B γ (eukaryotic translation elongation factor gamma-subunit), DHAR (dehydroascorbate reductase), TCHQD (tetrachlorohydroquinone dehalogenase), metaxin, and mPGES-2 (microsomal prostaglandin E synthase type 2) [1]. While zeta and theta classes are ubiquitous across organisms, lambda, tau, and DHAR classes are plant specific. Although phi classes were previously considered plant specific, homologous sequences have been identified in fungi, bacteria, and protists. The predominance of tau and phi classes in plants suggests their primary role in stress response.

Plant *GSTs* gained prominence due to their protective role against oxidative damage and stress conditions, facilitated by their broad substrate specificity [11,12]. Tau and phi *GSTs* exhibit GSH-dependent peroxidase activity (GPX, EC 1.11.1.9), catalyzing the conversion of hydrogen peroxide to water and organic hydroperoxides to alcohols, with concurrent formation of oxidized glutathione (GSSG) [9,11,13]. Theta class *GSTs* also demonstrate significant GSH-dependent peroxidase activity and are implicated in reducing oxidative stress metabolites, including secondary metabolites and fatty acid hydroperoxides [14,15,16]. DHAR class *GSTs* participate in the ascorbic acid/GSH cycle, where ascorbate peroxidase utilizes ascorbic acid to reduce hydrogen peroxide to monodehydroascorbic acid, which DHAR *GSTs* subsequently reduce to ascorbic acid using GSH as a substrate [17,18]. Beyond their catalytic functions, plant *GSTs* exhibit non-enzymatic activities through interactions with secondary metabolites, facilitating the transport of lipoproteins, anthocyanins, phenolics, and phytohormones. These interactions contribute to the degradation of harmful compounds and stress-signal transmission [10,18]. Additionally, zeta *GSTs* catalyze GSH-dependent isomerization reactions and participate in tyrosine catabolism [19].

*GSTs* in both plants and animals are associated with diverse cellular processes, including signal transduction, oxidative stress response, redox homeostasis regulation, apoptosis control, and secondary metabolite biosynthesis and transport [20,21,22,23]. In plants, *GSTs* are particularly crucial for drought-stress resistance, suggesting that modulation of *GST* expression and their regulatory pathways could enhance stress tolerance. Recent advances in plant epigenetics, particularly DNA methylation studies, have provided insights into drought-stress signal transduction mechanisms. While *GST* research in crops has primarily focused on gene cloning and functional validation, studies of epigenetic regulation in potato under drought stress remain limited. Previous studies documented the genome-wide characterization of the *GST* family of genes Doubled Monoploid (DM) potato [24]. The genome assembly of autotetraploid potato varieties (Collaboration 88, C88) was recently completed [25]; however, comprehensive analysis of the *GST* gene family in autotetraploid potatoes remains to be explored. This study presents a systematic analysis of autotetraploid *StGSTs*, encompassing subfamily classification, nomenclature, structural analysis, chromosomal distribution, sequence relationships, phylogenetic analysis, and expression patterns under drought stress and DNA demethylation, providing a foundation for understanding *StGST* function and developing stress-resistant potato varieties.

## 2. Materials and Methods

### 2.1. Genome-Wide Identification of Gst Genes in Potato C88

The potato C88 genome data were obtained from the Spud DB database (http://spuddb.uga.edu/, accessed on 5 August 2024). *GST* gene identification was performed using Hidden Markov Models (HMMs) for GST_C (PF00043) and GST_N (PF02798) domains downloaded from the Pfam database [26]. The C88 proteome was screened using HMMER v3.4 [27] with an E-value threshold of ≤1 × 10^−5^. Additionally, BLASTp analysis was performed against *Arabidopsis GST* (*AtGST*) protein sequences using the same E-value cutoff [28]. Results from both approaches were combined, and redundant sequences were removed. Domain structures were verified using Pfam, yielding 366 *GST* genes. Physicochemical properties were predicted using ExPASy (http://web.expasy.org/protparam/, accessed on 5 August 2024) [29], and subcellular localization was determined using WoLF PSORT II (https://www.genscript.com/wolf-psort.html?src=leftbar, accessed on 5 August 2024) [30].

### 2.2. Phylogenetic Analysis and Classification

Protein sequences of 61 *AtGST* genes were retrieved from UniProt (https://www.uniprot.org/, accessed on 7 August 2024). Multiple sequence alignment of C88 and *Arabidopsis GST* proteins was performed using MUSCLE in MEGA X v10.2.6. Phylogenetic trees were constructed using the Neighbor-Joining method with 1000 bootstrap replicates, employing the Poisson correction model and pairwise deletion. Tree visualization and subfamily classification were performed using Evolview (https://evolgenius.info/helpsite/qst1.html, accessed on 7 August 2024).

### 2.3. Motif and Structural Analysis

Protein motif analysis of C88 *GST* family members was conducted using MEME (http://meme-suite.org/tools/meme, accessed on 10 August 2024) with a maximum motif number of 10 and default parameters [31]. Domain analysis was performed using NCBI CDD (https://www.ncbi.nlm.nih.gov/cdd/, accessed on 10 August 2024) [32]. Exon–intron structures were determined using GFF (General Feature Format) data. TBtools v2.056 were used to integrate and visualize phylogenetic relationships, motif patterns, domain structures, and gene architectures.

### 2.4. Chromosomal Distribution and Evolutionary Analysis

*StGST* genes were mapped to C88 chromosomes using TBtools. Gene density was calculated using 200-kb windows and visualized using a blue (low) to red (high) gradient, with blank regions indicating unmapped genomic segments. Comparative synteny analysis was performed between autotetraploid potato C88 and five species (*Arabidopsis thaliana* L., *Oryza sativa* L., *Solanum tuberosum DM* L., *Solanum lycopersicum* L., and *Capsicum annuum* L.).

### 2.5. Promoter Analysis

Promoter sequences (2000 bp upstream) of *StGST* genes were extracted using TBtools and analyzed for cis-regulatory elements using PlantCARE (http://bioinformatics.psb.ugent.be/webtools/plantcare/html/, accessed on 15 August 2024) [33]. Element distribution was visualized using TBtools.

### 2.6. Expression Analysis Under Drought Stress

Potato cultivars Qingshu 9 (QS9, drought resistant) and Atlantic (ATL, drought sensitive) were obtained from Gansu Agricultural University. Single-node stem segments were cultured in MS liquid medium under controlled conditions (22 ± 2 °C, 25.0–37.5 μmol m⁻^2^s⁻^1^, 16 h photoperiod). After 24 days, plantlets were treated with 200 mM mannitol and 60 μM 5-aza-deoxycytidine (5-Aza-dC, Sigma-Aldrich, St. Louis, Missouri, USA). Stem and leaf samples were collected at 0, 2, 6, 12, and 24 h post-treatment, flash-frozen in liquid nitrogen, and stored at −80 °C. Three biological replicates were maintained per treatment.

Total RNA was extracted using the Tiangen RNA extraction kit (TIANGEN Biotech (Beijing) Co., Ltd., Beijing, China), quality-assessed by electrophoresis, and quantified spectrophotometrically. cDNA was synthesized using the TOYOBO reverse transcription kit (TOYOBO CO., LTD. Life Sciences Department, Osaka, Japan). Gene-specific primers were designed with potato actin as internal reference. Primer sequence is shown in Supplementary Table S1. qPCR reactions (20 μL) contained 1 μL cDNA, 10.4 μL SYBR Premix Ex Taq (2×), 1.6 μL primers, and 7 μL ddH_2_O. Amplification conditions were: 95 °C for 3 min; 35 cycles of 95 °C for 30 s, 58 °C for 30 s, and 72 °C for 30 s. Relative expression was calculated using the 2^−ΔΔCt^ method [34].

### 2.7. Protein Interaction Network and Functional Enrichment

*StGST* protein sequences were analyzed using the STRING database (https://string-db.org/, accessed on 17 August 2024) to predict protein–protein interactions based on *Arabidopsis* ortholog data. Interacting proteins were functionally characterized through GO and KEGG pathway enrichment analyses.

### 2.8. Transient Expression in Nicotiana Benthamiana

*Nicotiana benthamiana* plants were grown under controlled greenhouse conditions (22 ± 2 °C, 12 h photoperiod, 60% relative humidity). After 45 days, uniformly developed plants were selected and acclimated in Hoagland’s solution for 2 days prior to transformation. Agrobacterium tumefaciens-mediated transformation was performed using leaf infiltration, with empty *pC2300s* vector serving as the negative control. Two days post-transformation, plants were exposed to 200 mM mannitol and 60 μM 5-Aza-dC in Hoagland’s solution. Samples were collected after 6 h of treatment. Protein localization was visualized using confocal laser scanning microscopy(LSCM800 from Carl Zeiss AG located in Oberkochen, Germany).

### 2.9. Physiological Parameters Measurements

Physiological parameters (proline content (Pro), superoxide dismutase (SOD), peroxidase (POD), and catalase (CAT) activities) were measured with three biological replicates per assay. Each sample consisted of 0.2 g of frozen leaves, which were thoroughly homogenized in 2 mL of ice-cold 50 mM potassium phosphate buffer (pH 7.8) supplemented with 5 mM EDTA, 2 mM ascorbic acid, and 2% (*w*/*v*) polyvinyl pyrrolidone. The homogenate was centrifuged at 12,000 rpm for 10 min at 4 °C, and the supernatant was used as the crude extract for subsequent antioxidant enzyme activity assays.

The SOD activity was determined using the Nitrotetrazolium Blue chloride (NBT) method [35]. The assay medium was composed of 50 mM potassium phosphate buffer (pH 7.8), 1 mM EDTA, 130 mM methionine, 750 µM NBT, and 20 µM riboflavin. The reaction mixture was exposed to 4000 lux light for 20 min, and the absorbance was recorded at 560 nm.

The POD activity was determined using the Guaiacol method [36]. The reaction mixture comprised 200 mM potassium phosphate buffer (pH 7.0), 5.485 mM H_2_O_2_, 3.46 mM Guaiacol, and the crude enzyme extract. Absorbance changes were monitored at 470 nm.

The CAT activity was determined by UV absorption method [37]. The reaction mixture consisted of 50 mM potassium phosphate buffer (pH 7.0), 67 mM H_2_O_2_, and the crude enzyme extract. The activity of CAT was quantified by monitoring the decrease in the absorbance of hydrogen peroxide at 240 nm over a 2 min period.

Pro-content was determined using the acid ninhydrin method [38]: Cut 0.2 g of frozen leaves into small pieces and boil them in 5 mL of 3% (*w*/*v*) sulfosalicylic acid in a boiling-water bath for 10 min. Transfer 2 mL of the supernatant to a test tube and add 2 mL of 2.5% acidic ninhydrin reagent and 2 mL of glacial acetic acid. Boil the mixture in a boiling-water bath for 30 min and then cool to terminate the reaction. Extract the formed chromophore with 4 mL of toluene. Aspirate the toluene layer and centrifuge it at 3000 rpm for 5 min. Finally, measure the absorbance of the organic layer at a wavelength of 520 nm.

## 3. Results

### 3.1. Characterization of GST Gene Family in Potato C88 Genome

We identified 366 *GST* genes (*StGSTs*) in the potato C88 genome using data from the Spud DB database (http://spuddb.uga.edu/, accessed on 5 August 2024). Analysis of physicochemical properties and subcellular localization revealed substantial diversity among family members (Table S2). The predicted proteins ranged from 100 to 902 amino acids (09H1G010720–09H3G070630) with a mean length of 246 aa. Molecular weights varied from 11.304 to 103.295 kDa (05H2G041460–09H3G070630), averaging 28.36 kDa. Theoretical isoelectric points (pI) ranged from 4.12 to 9.71 (05H2G041460–08H1G022650), with most proteins exhibiting acidic characteristics (pI < 7). Subcellular localization analysis predicted that the majority of *StGSTs* localize to the cytoplasm (193/366) and chloroplasts (127/366), with smaller numbers in the nucleus (35/366), peroxisomes (6/366), mitochondrial matrix (3/366), and endoplasmic reticulum (2/366).

### 3.2. Phylogenetic Analysis and Classification of StGSTs

**Figure 1:**
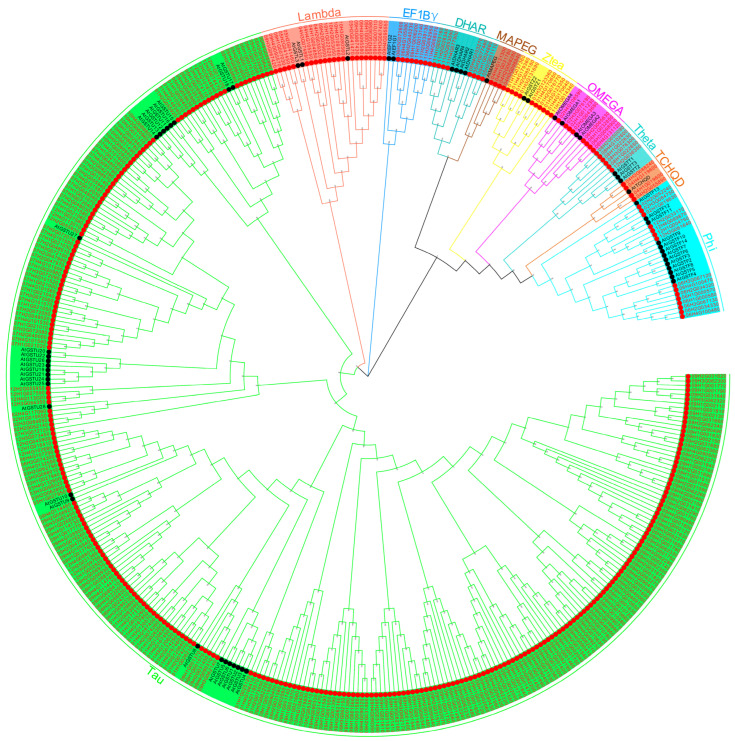
A phylogenetic tree of the relationship between *StGSTs* protein and *AtGSTs*, with Phi, TCHQD, Theta, OMEGA, Zeta, MAPEG, DHAR, EF1By, Lambda, Tau representing different subfamilies. Red fonts denote potato *GSTs*, and black fonts represent the *Arabidopsis GSTs*. Different colors in the background signify distinct subfamilies.

Phylogenetic analysis of 366 *StGSTs* and 61 *Arabidopsis GSTs* (*AtGSTs*) using TBtools (1000 bootstrap replicates) revealed ten distinct subfamilies: Phi, TCHQD, Theta, OMEGA, Zeta, MAPEG, DHAR, EF1Bγ, Lambda, and Tau (Figure 1). The Tau subfamily was the largest, comprising 281 members, followed by Lambda (22), Phi (15), Zeta (10), EF1Bγ, DHAR, and OMEGA (8 each), Theta (6), and MAPEG and TCHQD (4 each). Close clustering of multiple *StGSTs* with *AtGSTs* suggests high sequence homology, functional similarity, and evolutionary conservation.

### 3.3. Gene Structure and Conserved Motif Analysis of StGST Genes

To investigate the evolutionary diversity of the *StGST* gene family, the conserved motifs of 366 *StGST* proteins were analyzed using MEME, identifying 10 distinct conserved motifs (labeled motif 1–10) (Supplementary Figure S1A). Visualization with TBtools showed that genes within the same phylogenetic branch shared similar motifs, indicating potential functional similarities. The Tau subfamily displayed the highest number of conserved motifs, ranging from 2 to 8, with nearly all members containing motifs 1–5 and 7–8. Notably, the gene 09H3G062150 had 8 conserved motifs. In contrast, the Lambda subfamily contained 2–4 motifs, while the EF1By subfamily exhibited a unique motif (motif 10). The Omega subfamily contained only motif 6, while MAPEG (e.g., 05H2G041460) and Zeta (e.g., 01H3G123360) subfamilies contained motif 5 and motif 6, respectively. The Theta subfamily (e.g., 12H2G036350) did not exhibit any conserved motifs. Motif annotation revealed that motifs 1 and 4 were associated with GST_N domains, whereas motifs 2, 8, 9, and 10 were associated with GST_C domains. Domain analysis indicated that most Tau, Phi, and EF1By subfamily members contained both GST_N and GST_C domains, while other subfamilies had only one type of domain (Figure S1B).

Gene structure analysis revealed that members of the Tau, EF1By, Omega, TCHQD, and Phi subfamilies typically had 2–3 exons. In contrast, MAPEG and DHAR subfamily members had 5–8 exons, while Lambda and Theta subfamilies contained 8–10 exons. Most *StGST* genes contained 2 introns; however, the Theta subfamily gene 08H1G022650 contained 10 introns (Figure S1C). The distribution of exons and introns was consistent within subfamilies but varied significantly across subfamilies, suggesting structural divergence during evolution.

### 3.4. Analysis of Cis-Regulatory Elements in StGST Promoter Regions

Promoter sequences (2000 bp upstream) of *StGST* genes in potato C88 were analyzed using PlantCARE (http://bioinformatics.psb.ugent.be/webtools/plantcare/html/, accessed on 15 August 2024), and element distribution was visualized using TBtools (Figure S2). *StGST* promoters contained four major categories of cis-elements: hormone-responsive elements (ABRE, TGACG-motif, CGTGA-motif, TCA-element, and GARE-motif), stress-response elements (MBS, LTR, TC-rich repeats, and WUN-motif), metabolism-related elements (ARE, MBSI, and O2-site), and tissue-specific elements. The meristem-associated CAT-box was most abundant (15,272), followed by ABA-responsive elements (ABRE, 1126), antioxidant-responsive elements (ARE, 619), MeJA and salicylic acid-responsive elements (CGTGA-motif and TCA-element, 444), wound-responsive elements (WUN-motif, 387), and drought-responsive elements (MBS, 202) (Table S3).

### 3.5. Chromosomal Distribution and Duplication Analysis of StGST Genes

The 366 *StGST* genes showed non-uniform distribution across potato chromosomes, with the highest density on chromosome 9 (Figure S3). Genes within subfamilies exhibited random chromosomal distribution, predominantly clustering at chromosome termini. Using MCScanX v2022 and Circos v0.69-9 tools, we analyzed segmental and tandem duplications, identifying 13 tandem duplication events (defined as ≥2 genes within 200 kb): 01H3G123350/01H3G123360, 01H4G170640/01H4G170650, 03H1G027940/03H1G027950, 05H1G006990/05H1G007000, 05H1G007000/05H1G007010, 06H1G000570/06H1G000580, 07H2G048030/07H2G048040, 07H3G072090/07H3G072100, 07H4G101560/07H4G101570, 07H4G101580/07H4G101590, 09H1G001750/09H1G001760, 09H1G017760/09H1G017770, and 09H4G105940/09H4G105950. These duplications involved 25 genes across chromosomes 1, 3, 5, 6, 7, and 9, comprising nine pairs from the Tau subfamily, two from Zeta, and one from Phi. These findings suggest that tandem duplications significantly contributed to *StGST* family expansion during evolution.

### 3.6. Comparative Genomic Analysis of GST Proteins Across Species

**Figure 2:**
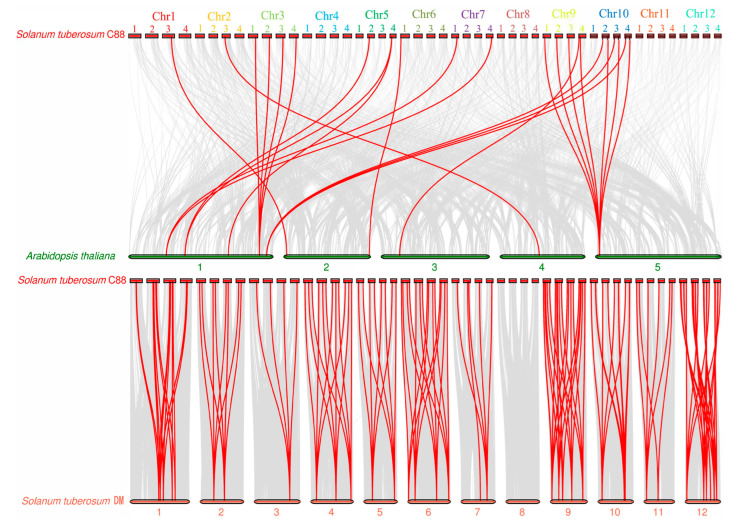
Syntenic relationships of *GST* genes between potato C88 and two species. Red lines highlight orthologous *GST* gene pairs between *Solanum tuberosum C88* and *Arabidopsis thaliana* and *Solanum tuberosum DM*.

To investigate evolutionary relationships of *GST* proteins, we conducted synteny analysis between autotetraploid potato C88 and five species: *Arabidopsis thaliana* (dicot), *Oryza sativa* (monocot), and three Solanaceae species (*Solanum tuberosum DM*, *Solanum lycopersicum*, and *Capsicum annuum*). The analysis revealed conserved syntenic relationships of 366 *StGSTs* across these species, with varying numbers of orthologous pairs: 23 in *Arabidopsis*, 8 in rice, 187 in potato DM, 134 in tomato, and 110 in pepper (Table S4, Figure S4). The limited synteny between autotetraploid potato C88 and rice suggests that most *GST* genes emerged after monocot–dicot divergence (Figure 2). In contrast, the extensive synteny (>100 gene pairs) between autotetraploid potato C88 and other Solanaceae species indicates high conservation within this family.

### 3.7. Protein Interaction Network and Functional Enrichment Analysis

**Figure 3:**
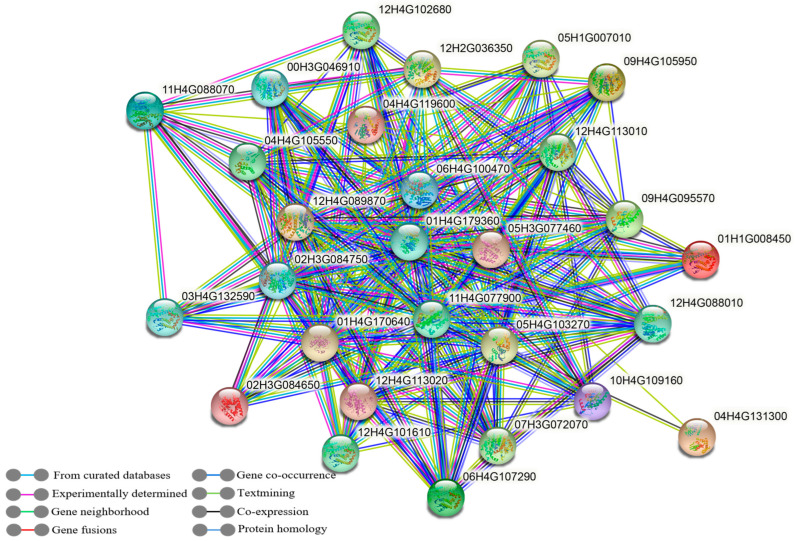
*StGST* protein interaction network. Nodes represent proteins and edges indicate protein–protein associations. Edge colors denote different types of interaction evidence. Minimum interaction confidence score: 0.400 (medium confidence).

Protein interaction analysis of *StGST* family members using STRING database revealed a network comprising 32 nodes and 207 edges (average node degree: 12.9; clustering coefficient: 0.588; PPI *p*-value < 1.0 × 10^−16^). GO enrichment analysis showed significant enrichment in biological processes (BP) and molecular functions (MF), with 177 genes involved in cellular and metabolic processes. KEGG pathway analysis revealed enrichment in glutathione metabolism (265 genes) and metabolic pathways (270 genes) (Table S5). Interacting proteins showed significant GO enrichment in stimulus response, cellular metabolism, cellular processes, and toxin response. Major enriched molecular functions included glutathione transferase and antioxidant activities. Cellular components were enriched in cytosol, cytoplasm, and cellular anatomical entities. KEGG analysis identified significant enrichment in glutathione metabolism, general metabolic pathways, ascorbate/aldarate metabolism, and tyrosine metabolism (Figure 3).

### 3.8. Expression Analysis of StGSTs Under Drought Stress and DNA Demethylation

**Figure 4:**
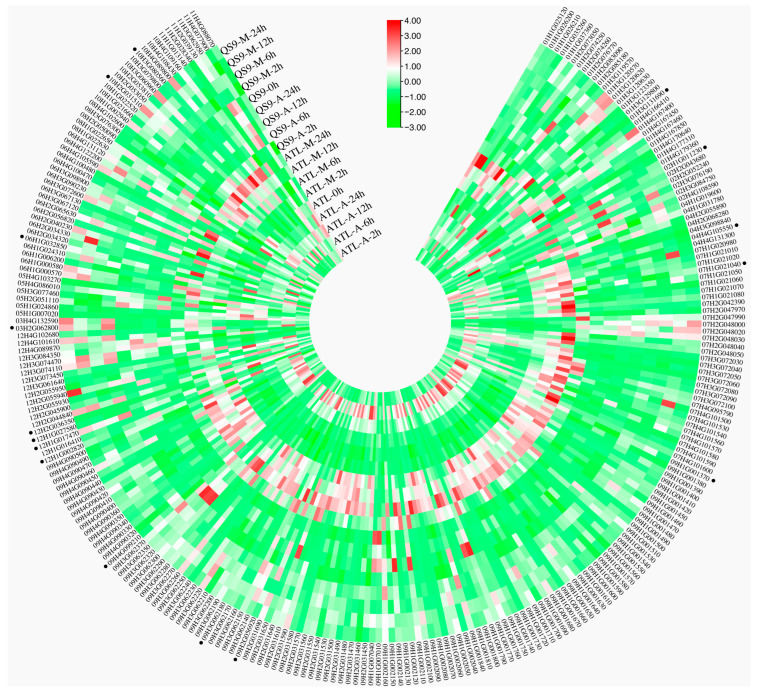
Transcriptional responses to drought stress and DNA demethylation. Heat map depicting expression patterns in drought-tolerant (QS9) and drought-sensitive (ATL) varieties under- treatment (M: Drought Stress; A: DNA Demethylation) after 0, 2, 6, and 12 h. Expression levels (FPKM values) were Z-score normalized. Color scale: green represents decreased expression; red represents increased expression. Black dots indicate the 16 genes that respond to both drought treatment and DNA demethylation.

**Figure 5:**
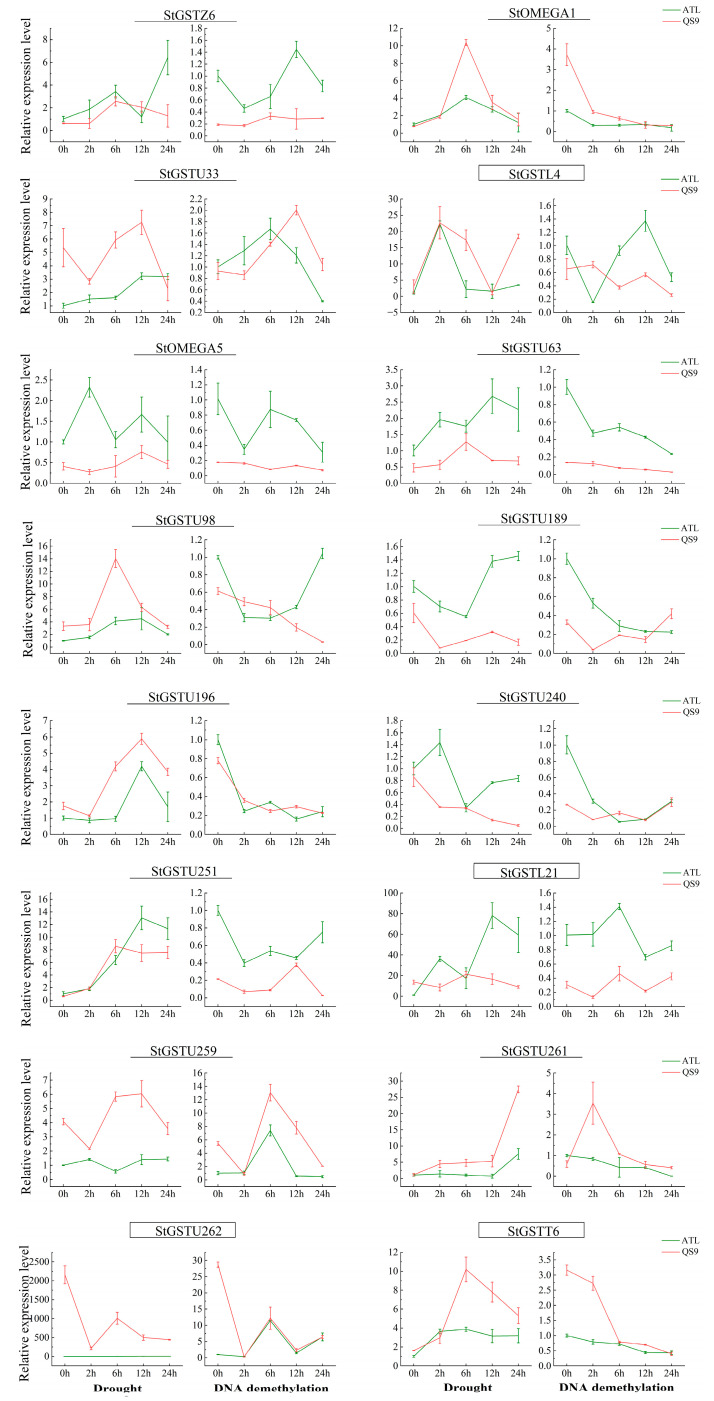
Gene-specific expression analysis of drought and DNA demethylation responsive *StGSTs*. Quantitative PCR analysis of 16 *StGST* genes showing dual responsiveness to drought stress and DNA demethylation in drought-tolerant (QS9) and drought-sensitive (ATL) varieties.

Previous transcriptomic analyses suggested that drought-stress responses of *StGST* genes might be regulated through DNA methylation pathways [39,40,41]. To investigate this relationship, we analyzed the transcriptome-wide expression (FPKM values) of all *StGST* family members in drought-tolerant (QS9) and drought-sensitive (ATL) potato varieties under both drought stress and DNA demethylation treatments. The two varieties exhibited distinct transcriptional responses to both treatments (Figure 4). From the 366 *StGST* members, we identified 16 genes that responded to both drought stress and DNA demethylation. Quantitative PCR validation demonstrated that four genes—*StGSTL4*, *StGSTT6*, *StGSTU262*, and *StGSTL21*—showed robust drought-stress responses, with expression levels exceeding a 10-fold increase. These genes also displayed significant responses to DNA demethylation treatment, with markedly different expression patterns between drought-tolerant QS9 and drought-sensitive ATL (Figure 5). Therefore, we selected these four genes as candidate genes for further study and highlighted their gene names with boxes in Figure 5. These differential responses warrant further investigation into the mechanistic relationship between drought resistance and DNA methylation regulation in these candidate genes.

### 3.9. Subcellular Localization of Candidate Genes

**Figure 6:**
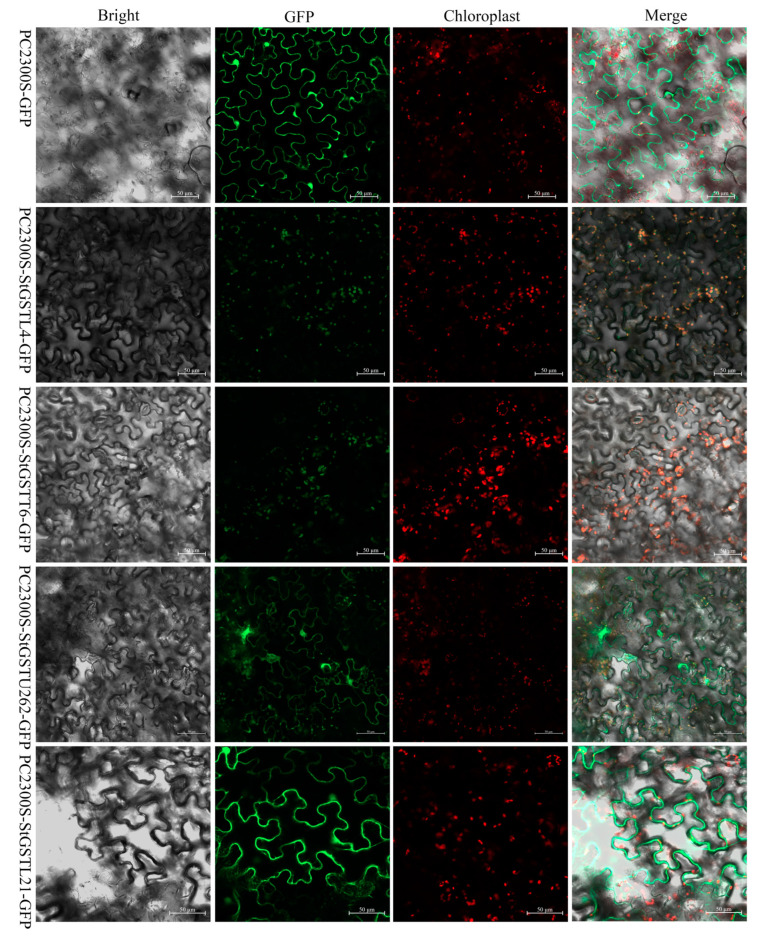
Subcellular localization of StGSTs-GFP-fusion proteins in tobacco leaves. Bright: bright field; GFP: green fluorescent protein; Chloroplast: chloroplast autofluorescence; Merge: the overlay of the first three images, Bright, GFP, and Chloroplast.

To determine the subcellular localization of four candidate genes, overexpression vectors *pC2300s-StGSTL4-GFP*, *pC2300s-StGSTT6-GFP*, *pC2300s-StGSTU262-GFP*, and *pC2300s-StGSTL21-GFP*, as well as the empty vector *pC2300s-GFP*, were introduced into the lower epidermis of tobacco leaves. Protein localization was observed using laser confocal microscopy. The empty pC2300s-GFP-fusion proteins vector localized to the nucleus, cell membrane, and cytoplasm. In contrast, pC2300s-StGSTL4-GFP-fusion proteins and pC2300s-StGSTT6-GFP-fusion proteins were specifically localized to chloroplasts, while pC2300s-StGSTU262-GFP-fusion proteins and pC2300s-StGSTL21-GFP-fusion proteins were detected in the cell membrane and cytoplasm (Figure 6). These results are consistent with predictions made by gene family analysis.

### 3.10. Candidate Genes Involved in Antioxidant Defense upon Drought Stress

To evaluate whether the four candidate genes contribute to drought resistance and DNA methylation regulation, their coding sequences (CDS) were cloned into vectors driven by the 35S promoter and transiently transformed into tobacco plants. Plants were treated with 200 mmol·L⁻^1^ mannitol or 60 μmol·L⁻^1^ DNA methylation inhibitor (5-Aza-dC) for 6 h. Stress-related physiological indices, including catalase (CAT), peroxidase (POD), superoxide dismutase (SOD) activities, and proline (Pro) content, were measured.

**Figure 7:**
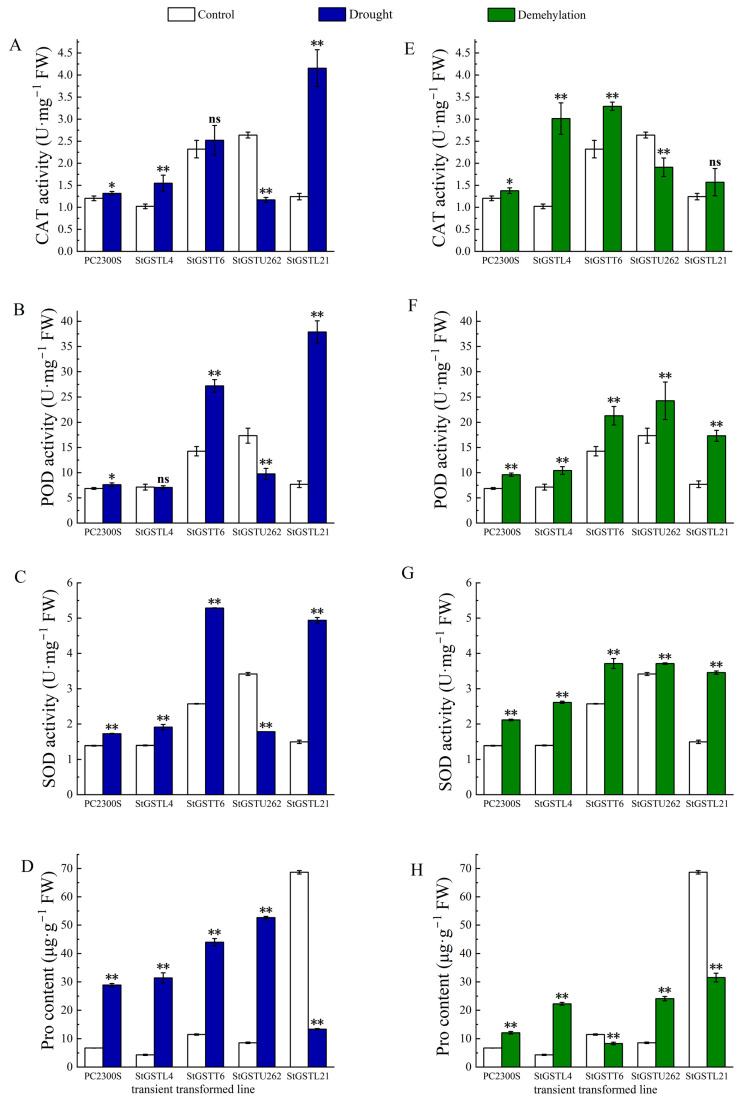
Physiological characteristics of transiently transformed tobacco under drought and demethylation treatments. (**A**,**E**) Catalase activity; (**B**,**F**) peroxidase activity; (**C**,**G**) superoxide dismutase activity; (**D**,**H**) proline content. Significant differences from the control are indicated, “ns” represents no significant difference. (* *p* < 0.05; ** *p* < 0.01, *t*-test).

Under drought treatment, the CAT activity of tobacco transformed with the empty *pC2300s* vector increased by 9.39% (*p* < 0.05). In comparison, CAT activity significantly increased by 51.45% and 234.30% in *StGSTL4* and *StGSTL21*-transformed plants, respectively, but decreased by 55.71% in *StGSTU262*-transformed plants. *StGSTT6* showed no significant change (*p* < 0.05) (Figure 7A). For POD activity, the empty vector increased activity by 10.87% under drought stress. *StGSTT6* and *StGSTL21* significantly increased POD activity by 90.10% and 391.86%, respectively, while *StGSTU262* showed a 43.74% decrease (*p* < 0.05) (Figure 7B). Similarly, SOD activity increased by 24.32% in the empty vector, with significant increases of 34.20%, 105.55%, and 230.86% in *StGSTL4*, *StGSTT6*, and *StGSTL21*-transformed plants, respectively. In contrast, *StGSTU262*-transformed plants showed a significant decrease of 47.83% (*p* < 0.05) (Figure 7C). Proline content in empty-vector-transformed plants increased by 330.82% under drought stress. *StGSTL4* and *StGSTU262* significantly increased proline levels by 629.72% and 517.53%, respectively, while *StGSTL21* decreased by 80.19% (*p* < 0.05) (Figure 7D).

Under DNA demethylation treatment, CAT activity increased by 14.42% in the empty vector. *StGSTL4*, *StGSTT6*, and *StGSTL21* increased CAT activity by 198.15%, 41.84%, and 26.34%, respectively, while *StGSTU262* decreased by 27.63% (*p* < 0.05) (Figure 7E). POD activity increased by 39.78% in empty vector plants. Significant increases of 46.23%, 49.42%, and 150.84% were observed in *StGSTL4*, *StGSTT6*, and *StGSTL21*-transformed plants, respectively (*p* < 0.05) (Figure 7F). For SOD activity, the empty vector showed a 52.41% increase, with *StGSTL4* and *StGSTL21* showing increases of 87.52% and 131.56%, respectively. *StGSTT6* showed no significant change, while *StGSTU262* decreased by 20.65% (*p* < 0.05) (Figure 7G). Proline content in empty vector plants increased by 80.39%. *StGSTL4* and *StGSTU262* significantly increased proline levels by 417.51% and 182.68%, respectively, while *StGSTT6* and *StGSTL21* decreased by 27.53% and 54.08% (*p* < 0.05) (Figure 7H).

Overall, the results indicate that the four candidate genes enhance antioxidant enzyme activities and osmotic regulation under drought and demethylation treatments, suggesting a positive role in drought resistance potentially mediated by DNA methylation. Further studies are required to elucidate the underlying mechanisms.

## 4. Discussion

### 4.1. Identification of Gene Family Members

The glutathione S-transferase (GST) family plays a crucial role in plant responses to abiotic stress [42,43]. Using comprehensive bioinformatics analyses, we identified 366 *StGST* gene family members in the potato C88 genome and characterized their physicochemical properties (Table S2). The encoded proteins range from 100 to 902 amino acids (09H1G010720–09H3G070630), with an average length of 246 amino acids. Their relative molecular weights vary from 11.304 to 103.295 kDa (05H2G041460–09H3G070630), averaging 28.36 kDa. These structural variations suggest evolutionary divergence in response to environmental pressures. Most *StGST* proteins exhibit a pI < 7, indicating enrichment in acidic amino acids—a characteristic shared with other dicotyledonous plants but contrasting with monocotyledonous plants, reflecting their evolutionary divergence. Through transient expression in tobacco, we demonstrated that StGSTL4 and StGSTT6 fusion proteins localize to chloroplasts, while StGSTU262 and StGSTL21 fusion proteins localize to cell membranes and cytoplasm (Figure 6), consistent with previous findings [44,45].

### 4.2. Gene Structure Analysis

Following the *Arabidopsis GST* classification system, we categorized the *366 StGSTs* into ten subfamilies: Phi, TCHQD, Theta, OMEGA, Zeta, MAPEG, DHAR, EF1By, Lambda, and Tau (Figure 1). Phylogenetic analysis revealed close clustering between multiple *StGSTs* and *AtGSTs*, suggesting high homology, functional similarity, and evolutionary conservation. While physicochemical properties vary considerably among *StGST* family proteins, gene structures and amino acid motifs remain relatively conserved within subfamilies (Figure S1). Gene structure analysis provides crucial insights into evolution and amplification patterns [46]. Chromosomal rearrangements and duplications can lead to exon/intron gains or losses, contributing to gene family diversification [47,48]. The identified *StGSTs* are rich in coding sequences (CDS) and untranslated regions (UTRs), with subfamily members sharing similar conserved motifs, domains, and exon–intron structures. However, these features differ substantially between subfamilies, indicating distinct functional roles [49,50]. Some subfamily members, such as 09H3G070630 (Tau) and 08H1G022650 (Theta), possess unique structural elements, suggesting functional specialization. Based on subfamily size and intron patterns, we hypothesize that the Tau class represents the ancestral *GST* subfamily in potatoes. The widespread distribution of motif 1 across *StGSTs* suggests it may be a defining feature of this gene family.

### 4.3. Chromosomal Distribution and Comparative Genomic Analysis

The variation in gene family size across species likely results from evolutionary processes including gene recombination, duplication events, and species-specific differentiation [51,52]. Plant *GST* family expansion primarily occurs through serial duplication of tau and phi classes, making these plant-specific subfamilies the largest [48,53,54]. Our analysis revealed that the 366 *StGST* genes are unevenly distributed across the 12 potato chromosomes, with subfamily members dispersed randomly. Chromosome 9 harbors the highest concentration of *StGST* genes, indicating chromosome-specific expression patterns (Figure S3). We identified 13 tandem duplication events involving 25 *StGST* genes on chromosomes 1, 3, 5, 6, 7, and 9. The majority of these duplicated genes belong to the Tau subfamily, suggesting coordinated protein expression and regulatory functions.

Comparative genomic analysis between potato C88 (autotetraploid) and *Arabidopsis*, rice, potato DM (Doubled Monoploid), tomato, and pepper revealed stronger syntenic relationships among dicotyledonous plants, reflecting their evolutionary history [51]. The limited collinearity between potato and rice (monocot) suggests that most *GST* genes emerged after dicot–monocot divergence. However, we observed over 100 syntenic gene pairs between autotetraploid potato C88 and its diploid relatives (potato DM, tomato, and pepper) (Figure S4), indicating high *GST* family conservation within the Solanaceae. These patterns of genome similarity and divergence illuminate species relationships and genome evolution, while suggesting strong selective pressure on the *StGST* gene family.

### 4.4. Promoter Cis-Regulatory Element and Protein Interaction Analyses

Promoter regions contain multiple cis-regulatory elements that serve as binding sites for transcription factors, playing crucial roles in environmental response regulation [55,56]. Analysis of the 366 *StGST* promoter sequences revealed diverse response elements related to abiotic stress and phytohormones, with meristem-specific elements being most abundant. The widespread distribution of hormone- and stress-responsive elements suggests extensive regulatory networks (Figure S2). Gene ontology (GO) enrichment analysis showed significant enrichment in biological processes (BP) and molecular functions (MF). KEGG pathway analysis revealed 265 genes significantly enriched in glutathione metabolic pathways associated with drought-stress response. GO analysis of GST-interacting proteins showed significant enrichment in stimulus response, cellular metabolic processes, cellular processes, and toxic substance response pathways, indicating coordinated roles in drought-stress resistance. These findings suggest that modulating these genes and their regulatory pathways could enhance plant drought tolerance.

### 4.5. Functional Characterization Under Drought Stress and DNA Demethylation

Previous studies have demonstrated the regulatory importance of *StGSTs* in abiotic stress responses. Transgenic studies have shown that overexpression of various *GST* genes (*OsGSTU4*, *LeGSTU2*, *ThGSTZ1*) enhances drought tolerance in *Arabidopsis* [57,58,59]. Loss of *AtGSTU17* function increases drought and salt tolerance [60]. Overexpression studies of Tau class *GSTs* from various species in tobacco have demonstrated enhanced *GST* activity and improved drought tolerance through multiple physiological mechanisms [58,61,62,63]. *JrGSTU1* gene in walnut (*Juglans regia* L.) can enhance GST activity to regulate the expression of other stress-related genes, thereby reducing the level of reactive oxygen species [64]. Overexpression of *MruGSTU39* in alfalfa (*Medicago sativa*) significantly improves the tolerance of transgenic plants to drought stress [65].

Abiotic stress can trigger DNA methylation changes, affecting stress-related gene expression [66]. For example, *TaGAPC1* DNA demethylation enhances drought stress response in resistant wheat varieties, while DNA methylation of *ZmNAC111* modulates maize drought tolerance [67,68]. In screening for osmotic stress-induced DNA methylation changes in maize using methylation sensitive amplification polymorphism (MSAP), a *ZmGST* was found to be induced by salt stress and undergo DNA demethylation, which resulted in up-regulation of its expression [69]. Our previous transcriptome analysis indicated potential regulation of *GST* expression through DNA demethylation under drought stress [40]. In this study, we identified 16 *StGST* genes showing significant responses to both drought and demethylation treatments, with differential expression between extremes of drought tolerance. Transient expression of four selected *StGST* genes in tobacco demonstrated that their overexpression significantly enhanced drought resistance. This enhancement operated through increased antioxidant defense and ROS-scavenging activity, suggesting positive roles in drought resistance potentially regulated through DNA methylation pathways, though specific molecular mechanisms require further investigation.

## 5. Conclusions

In this study, we conducted the first systematic identification of 366 *StGST* genes from the autotetraploid potato genome. These genes were classified into ten subfamilies (Phi, TCHQD, Theta, OMEGA, Zeta, MAPEG, DHAR, EF1By, Lambda, and Tau). While the physicochemical properties of the *StGST* family members showed considerable variation, their gene structures and protein motifs demonstrated high conservation within subfamilies. The *StGST* genes were distributed across all 12 potato chromosomes, with 13 tandem duplication events identified. Comparative genomic analysis revealed highest homology with other dicotyledonous plants. Cis-element analysis showed *StGST* genes particularly respond to abiotic stress. Subcellular localization studies demonstrated chloroplast localization for two StGST fusion proteins and cytoplasmic/membrane localization for two others. RNA-seq analysis revealed that most *StGSTs* responded to both drought stress and DNA demethylation treatments. Quantitative PCR validation of 16 selected *StGSTs* identified four members that showed strong responses to both treatments, with distinct expression patterns between drought-tolerant (QS9) and drought-sensitive (ATL) varieties. Functional characterization identified four *StGST* genes that responded strongly to both drought stress and DNA demethylation treatment, suggesting their positive role in stress tolerance. The underlying functional mechanisms still need to be verified and further analyzed in potato. These results provide a foundation for future investigations into the functions of *GSTs* in potato.

## Data Availability

All data are available within the manuscript.

## Supplementary Materials

The following supporting information can be downloaded at: https://www.mdpi.com/article/10.3390/antiox14020239/s1; Table S1: Primer sequences, Table S2: Characteristics of *GST* gene family members in potato (C88) genome, Table S3: Cis-element analyses of the *GST* gene promoter regions, Table S4: Covariant gene pairs, Table S5: Enrichment results statistics; Figure S1: *StGSTs* family member motif, domain, and gene structure; Figure S2: Cis-regulatory Elements in *StGST* Promoter Regions; Figure S3: Chromosomal Distribution of *StGST* Genes; Figure S4: Syntenic Relationships of *GST* Genes Between Potato C88 and Five Species.
