# Supplementary material for: Genome-Wide Identification and Drought-Responsive Functional Analysis of the GST Gene Family in Potato (Solanum tuberosum L.)
Source: Antioxidants (Basel). 2025 Feb 19;14(2):239. doi: 10.3390/antiox14020239 (PMC11852095; doi:10.3390/antiox14020239)
Supplement: Supplementary file 1 [file antioxidants-14-00239-s001.zip › Figure S1.pdf]

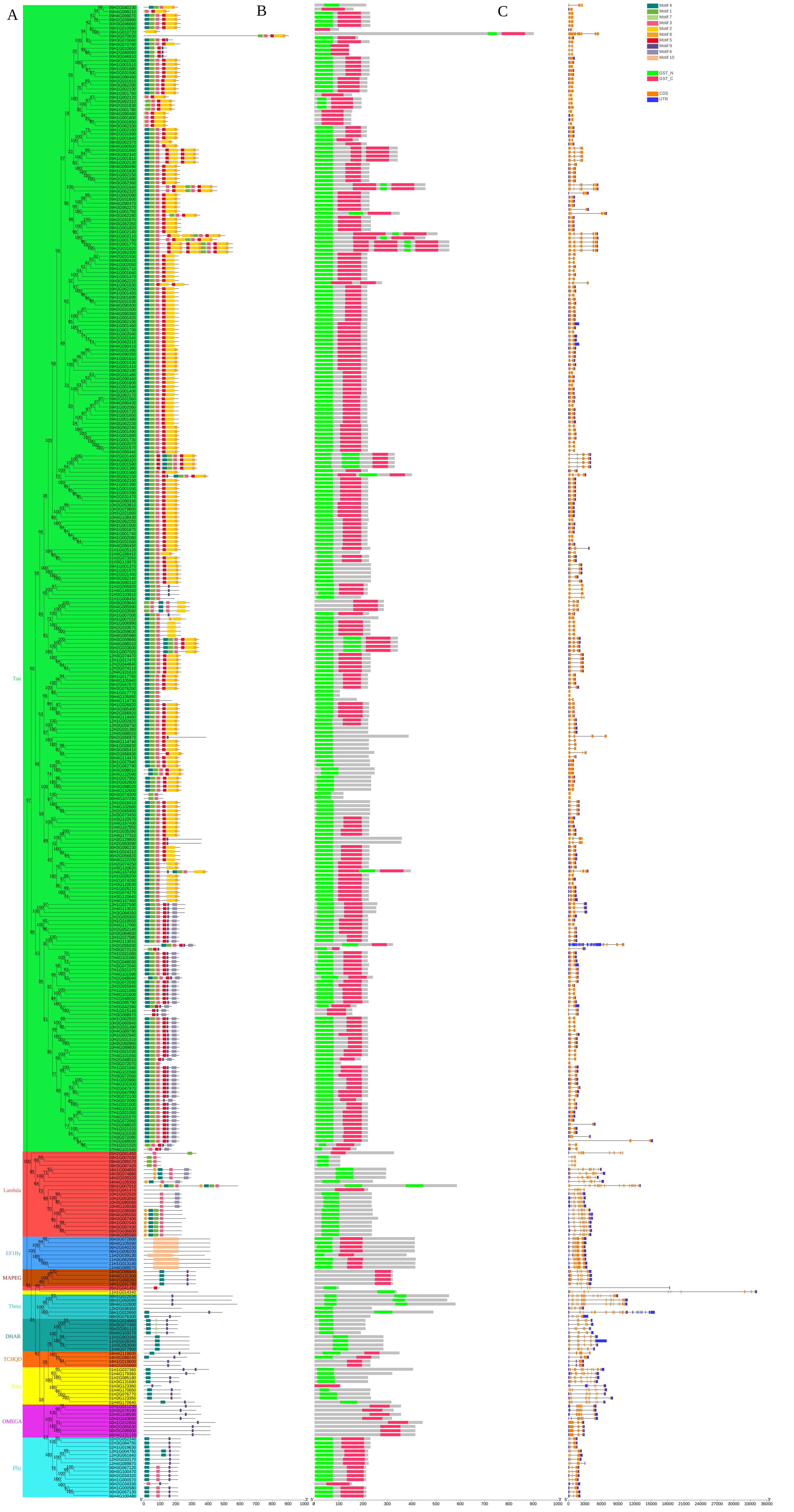

Figure S1. *StGSTs* family member motif, domain, and gene structure. (A)The phylogenetic tree was constructed using the full-length sequences of *StGST* proteins with 1000 replicates on each node. The amino acid motifs (numbered 1–10) in *StGST* proteins are displayed in ten colored boxes, and black lines indicate amino acid length. (B) Green represents the GST_N-terminal structural domain and pink rectangle represents the GST_C-terminal structural domain. (C) Blue rectangles and orange rectangles indicate UTR (non-coding region), CDS (coding sequence or exons).
