# Supplementary material for: Genome-Wide Identification and Drought-Responsive Functional Analysis of the GST Gene Family in Potato (Solanum tuberosum L.)
Source: Antioxidants (Basel). 2025 Feb 19;14(2):239. doi: 10.3390/antiox14020239 (PMC11852095; doi:10.3390/antiox14020239)
Supplement: Supplementary file 1 [file antioxidants-14-00239-s001.zip › Figure S2.pdf]

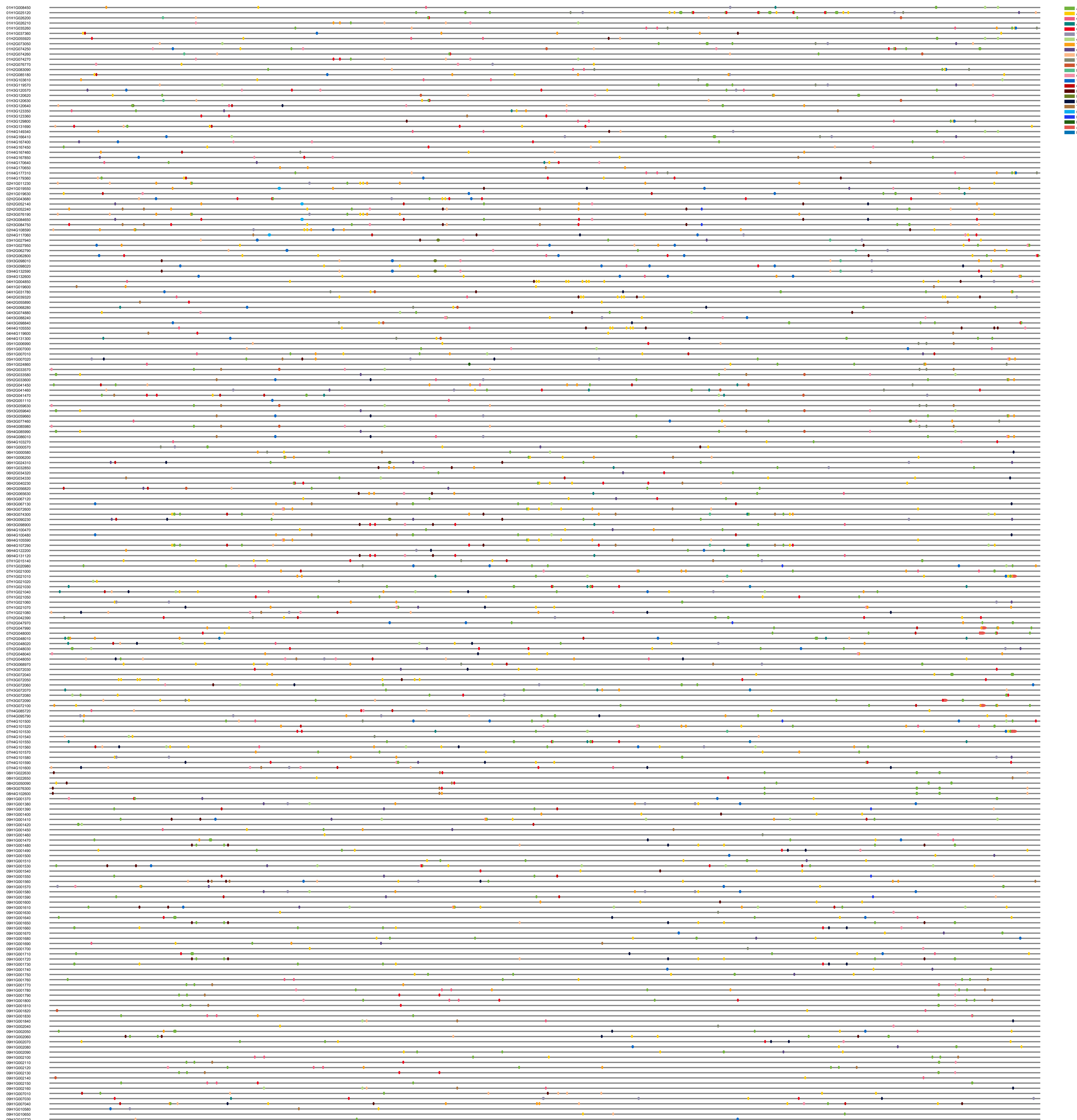

Figure S2. Cis-regulatory Elements in StGST Promoter Regions. ABRE: ABA-responsive element; ARE: anaerobic-responsive element; CAT-box: meristem-specific element; AuxRR-core: growth hormone core; TGACG-motif and CGTCA-motif: methyl jasmonate-responsive elements; TCA-element: salicylic acid-responsive element; circadian: circadian rhythms; GCN4_motif: con-trolling seed-specific expression of the genes studied. MBS: MYB binding site involved in drought response; WUN-motif: wound-responsive element; O2-site: zein metabolism regulatory element; TC-rich repeats: stress-responsive elements; GARE-motif: gibberellic acid-responsive element; LTR: low-temperature responsive element; TGA-element: regulation of plant resistance.
