# Supplementary material for: Genome-Wide Identification and Drought-Responsive Functional Analysis of the GST Gene Family in Potato (Solanum tuberosum L.)
Source: Antioxidants (Basel). 2025 Feb 19;14(2):239. doi: 10.3390/antiox14020239 (PMC11852095; doi:10.3390/antiox14020239)
Supplement: Supplementary file 1 [file antioxidants-14-00239-s001.zip › Figure S3A.pdf]

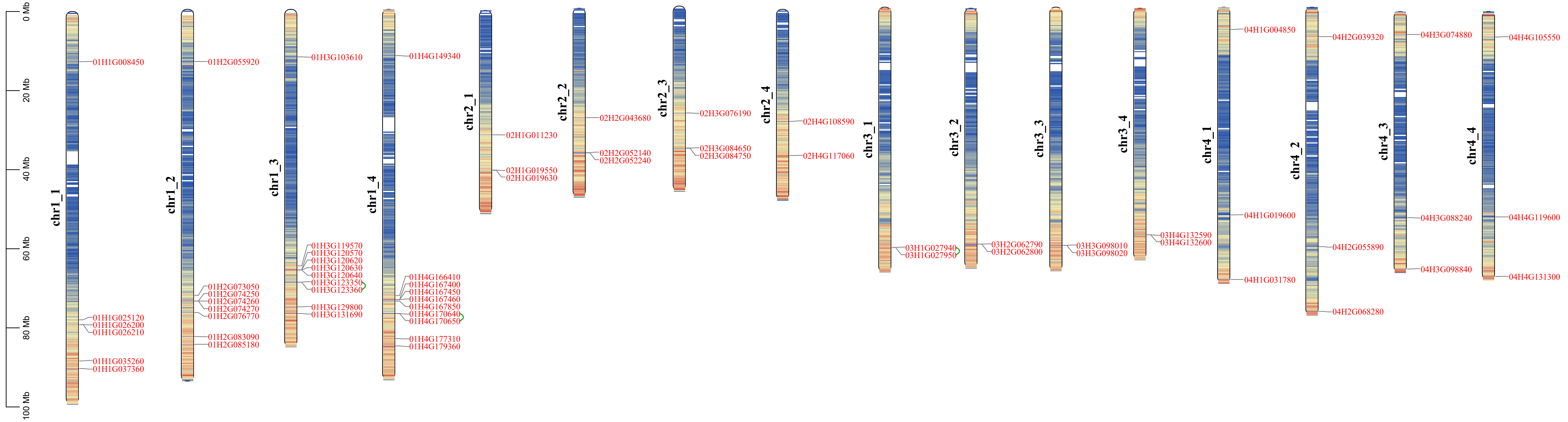

Figure S3A. Chromosomal Distribution of *StGST* Genes. Colored bars represent potato chromosomes (Chr 1-4); *StGST* menbers are shown in red text.
