# Supplementary figures and images for: Genome-Wide Identification and Drought-Responsive Functional Analysis of the GST Gene Family in Potato (Solanum tuberosum L.)
Source: Antioxidants (Basel). 2025 Feb 19;14(2):239. doi: 10.3390/antiox14020239 (PMC11852095; doi:10.3390/antiox14020239)

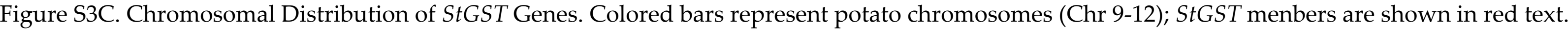

Supplement: Supplementary file 1 [file antioxidants-14-00239-s001.zip › Figure S3C.pdf]

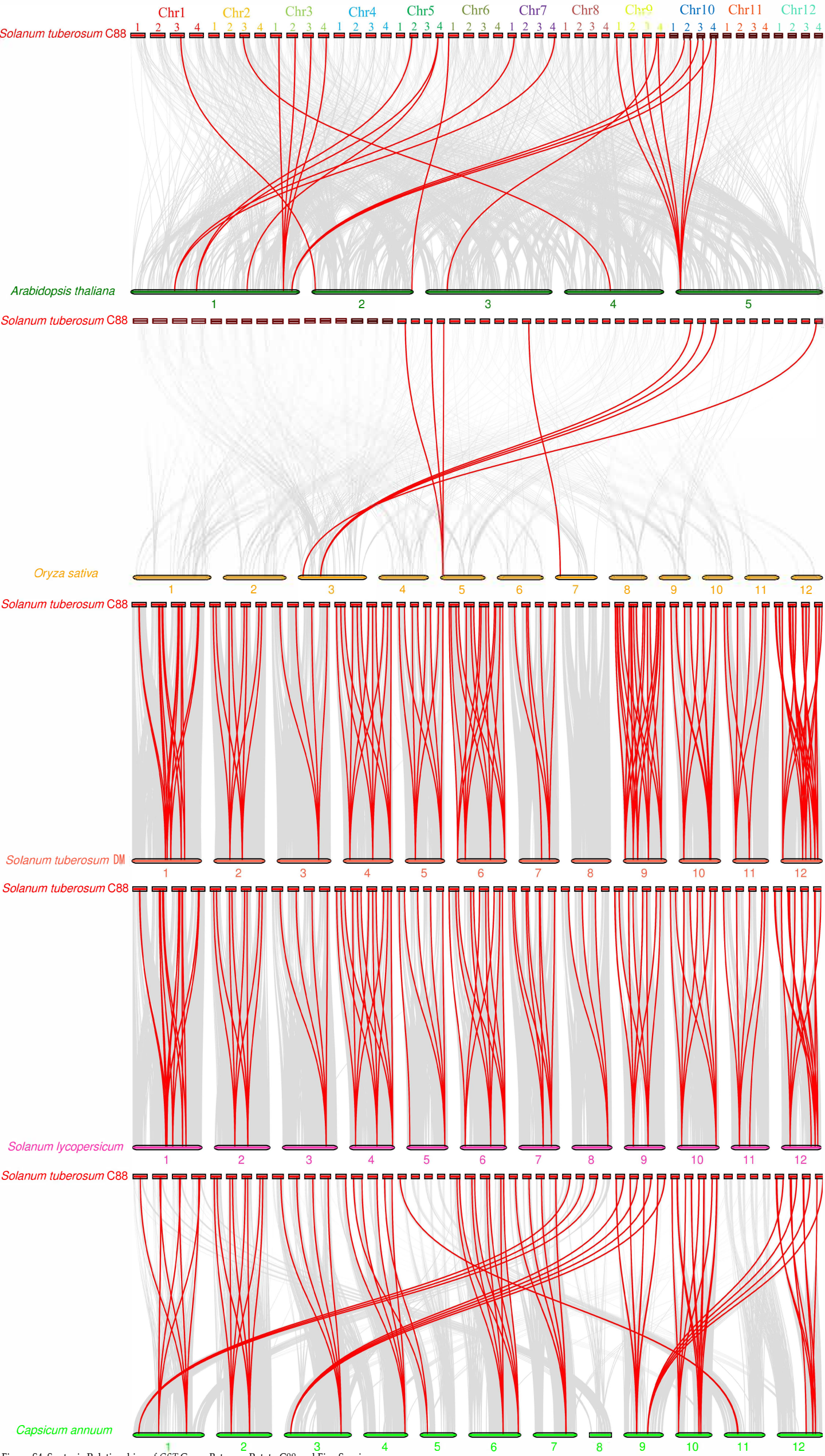

Figure S4. Syntenic Relationships of GST Genes Between Potato C88 and Five Species.

Supplement: Supplementary file 1 [file antioxidants-14-00239-s001.zip › Figure S4.pdf]
